# Shelter dog behavior after adoption: Using the C-BARQ to track dog behavior changes through the first six months after adoption

**DOI:** 10.1371/journal.pone.0289356

**Published:** 2023-08-16

**Authors:** Kyle R. Bohland, Meghan Leanne Lilly, Meghan E. Herron, Andréia G. Arruda, Jeanette M. O’Quin

**Affiliations:** 1 Department of Veterinary Clinical Sciences, College of Veterinary Medicine, The Ohio State University, Columbus, Ohio, United States of America; 2 Gigi’s Shelter for Dogs, Canal Winchester, Ohio, United States of America; 3 Department of Veterinary Preventive Medicine, College of Veterinary Medicine, The Ohio State University, Columbus, Ohio, United States of America; UFSJ: Universidade Federal de Sao Joao del-Rei, BRAZIL

## Abstract

Despite millions of dogs entering and exiting shelters annually, little is known about dog behavior long-term after adoption. Entering a shelter is stressful for dogs which may inhibit or exaggerate behavior. There is a common public sentiment that dogs have a “honeymoon period” after adoption where dogs do not show their full repertoire of behaviors, both positive and negative, until getting more comfortable in their new home. The aim of this prospective observational cohort study was to identify prevalence of and changes in behavior issues in dogs throughout the first six months following adoption. The owners of ninety-nine dogs adopted from five Ohio shelters between October 1, 2020 and June 1, 2021 were surveyed 7, 30, 90, and 180 days after adoption, using the Canine Behavioral Assessment & Research Questionnaire (C-BARQ). Owners were also asked about household changes that may affect behavior. Estimated age, sex, weight, length of shelter stay, shelter intake reason, use of gastrointestinal, antibiotic, and psychotropic medications in the shelter, whether the animal had been previously returned to the shelter, and whether the owner was a first-time dog owner, were evaluated as predictors in a mixed effect regression model of different behavior measures over time. At various timepoints, dogs showed high prevalence for stranger-directed aggression (81.7%), owner-directed aggression (32.3%), dog-directed aggression (75%), familiar dog aggression (37.8%), stranger directed fear (58.2%), nonsocial fear (95.8%), dog directed fear (80.0%) and separation-related behaviors (92.6%). Over 180 days, stranger-directed aggression, excitability, touch sensitivity, training difficulty, and chasing increased, while separation-related behaviors, attachment and attention-seeking decreased. Owners reported high satisfaction with their dogs’ behavior. Use of psychotropic medications in the shelter was predictive of stranger-directed aggression and touch sensitivity at home. These findings help veterinarians and shelter professionals council owners on potential behavior changes after adoption.

## Introduction

At least two million dogs are adopted from shelters in the United States every year [1]. Confinement in a shelter environment is stressful for dogs, resulting in measurable behavioral and physiologic changes [2, 3]. They experience a new environment with new scents, sounds, unfamiliar people, and unfamiliar animals [4]. They are also often separated from previous attachment figures. This stress can decrease welfare in shelter-housed dogs and contribute to behavior changes [5]. Once adopted, another stressful adjustment period begins. Veterinarians and shelter professionals often refer to this period as the “honeymoon period,” which can be a time of dynamic behavior changes. During times of stress and transition, a full picture of a dog’s behavior is not typically apparent until they are fully acclimated and comfortable in their new environment, which may be weeks or months after adoption [6, 7].

There is significantly more research on dog behavior while in the shelter compared to their behavior after adoption. Many shelters conduct formal behavior evaluations, however, the evidence is mixed whether these tools actually predict future behavior after adoption [7, 8]. This is particularly concerning because behavior problems have been consistently listed as the top reason for relinquishment of dogs to shelters and contribute to returns after adoption [9–14]. Previous post-adoption studies have largely relied on ad-hoc survey tools to track behavior, preventing any cross-study comparisons and contributing to significant variation in reported behavior changes. Additionally, only a few studies evaluating behavior changes after adoption tracked dogs at two timepoints in their new homes [15–18] and only one study could be identified with three timepoints [19].

When tracking aggression towards unfamiliar people and aggression in the veterinary setting, Stephen and Ledger found twice as many dogs worsened in those areas compared to those dogs who improved between two and six weeks after adoption [15]. Lord et al. found a similar number of owners reported behavior problems one week after adoption (63%) compared to four weeks after adoption (68%) [16]. Housetraining was the most reported behavior problem for dogs [16]. Incidence of aggression, (biting, growling, or snapping at people or animals) and problem behaviors when left alone increased slightly from one week to one month, while owners reporting shy, fearful, or hiding behaviors decreased slightly [16].

Outside the United States, Vitulová et al. found a statistically significant decrease in fearfulness in dogs adopted from a Czech Republic shelter six months after adoption compared to one week after adoption (decrease from 61% to 20%) [17]. There was no change in aggression across these two time points in this survey [17]. Gates et al. reported that 20% of dogs had an increase in the number of reported behavior problems, while 30% saw the number of behavior problems decrease or resolve during the study, but didn’t report specific behaviors for this population of 25 dogs [18]. Another challenge of Gates study was that the two time points of administering the survey were not consistent among dogs (between one week and six months depending on staff availability) hampering cross-study or inter-individual comparisons [18].

Attempts to compare shelter behavior to post-adoption behavior have widely varying findings. Mornement et al. used the Behavioural Assessment for Re-homing K9’s (B.A.R.K.) to assess dog behavior in five Australian shelters during their shelter stay and then again in the home an average of four months later (ranging from two to eight months post-adoption) [20]. Fear and anxiety in the shelter significantly predicted “fearful/inappropriate toileting” and “fear of strangers” post adoption, but no other scores in the shelter predicted behavior problems in the home [20]. At three months post-adoption from a shelter in Massachusetts, roughly half of dogs deemed food aggressive in the shelter showed the behavior at home and 22% of dogs deemed not food aggressive in the shelter later showed food aggression in their adoptive home [21]. At 13 months after adoption, 40.9% of dogs from a New York shelter exhibited lunging, growling, snapping, and/or biting [22]. Per the shelter policy, dogs that snarled, growled, lunged, snapped, or bit in any situation during the in-shelter standardized test besides resource guarding were euthanized, suggesting either the aggressive behaviors developed post-adoption or that these screening tools are potentially very limited, or both [22].

A recent study investigating owner expectation and owner return rates after adoption tracked dog behavior after adoption for three timepoints over four months [19]. Owners were contacted at two days, two weeks, and four months after adoption [19]. Prevalence of C-BARQ subscales were reported at each timepoint. Notably, at four months, 55.6% of dogs showed stranger-directed aggression and 85.2% showed separation-related behavior [19]. Friedman and Wilcoxon signed-rank tests were used to evaluate differences in C-BARQ subscales between time points and dog-directed aggression and nonsocial fear were found to significantly increase across timepoints [19].

To better characterize when and how behavior changes post adoption, this survey was conducted over four timepoints and is only the second study to use the standardized, validated C-BARQ to track dog behavior after adoption [19]. This research tracked newly adopted dogs to identify the prevalence and changes over time in several commonly reported behaviors. We also investigated owner satisfaction and specific dog and household characteristics as potential factors in post-adoption canine behavior changes.

## Materials and methods

### Participant recruitment and data collection

Five Ohio shelters collaborated in this study: an open access municipal shelter and four 501(c)(3) nonprofit shelters, two of which serve as the humane investigation departments for their respective counties. The municipal shelter adopted 3436 dogs in 2021, while the other four shelters adopted between 477 and 705 dogs that year. Four shelters were in the Columbus area and the fifth shelter was located in Cleveland.

Between October 1, 2020 and June 1, 2021, owners over 18 years of age were recruited at the time of adoption by shelter staff using a standard script (S1 File) and a flier containing a QR code to the survey registration cite. One researcher (KB) also occasionally visited the two closest shelters to assist with recruitment. Four of the shelters shared recruited adopters’ email addresses with the researchers weekly so a reminder email could be sent. Each dog’s unique identifier from the shelter was shared with the owner, utilized as a survey identifier, and reported to the investigators at the time of consent. Written consent was obtained online at the time of the first survey. Participants had to be >18 years of age, and all shelters had a policy of only adopting to those >18 years of age. Participation was voluntary and respondents could leave the study at any time by request or simply not responding to emailed surveys. The study protocol was reviewed and approved by the Institutional Review Board at the Ohio State University (protocol numbers: 2020E0575, 2020E1064, 2021E0078, and 2021E0233).

Once enrolled, survey data from owners were collected and managed using REDCap (S6 File) electronic data capture tools hosted at The Ohio State University [23, 24]. REDCap (Research Electronic Data Capture) is a secure, web-based software platform designed to support data capture for research studies providing 1) an intuitive interface for validated data capture; 2) audit trails for tracking data manipulation and export procedures; 3) automated export procedures for seamless data downloads to common statistical packages; and 4) procedures for data integration and interoperability with external sources.

Survey requests were emailed to owners at seven, 30, 90, and 180 days after adoption. Email reminders were sent by REDCap (if not yet completed) three and six days later. Adopters had seven days to complete each survey before it was closed to prevent completion of surveys outside of a narrow window. To encourage participation in the final survey, one team member (KB) called or text messaged owners three days after the 180-day survey was sent if not completed. No other contact (including behavioral or medical advice) was made with survey respondents by research team members. An incentive of two electronic gift cards from Amazon.com were utilized: $10 for completion of the first survey and $20 after completion of the fourth survey.

### Survey

The 42-question version of the C-BARQ was used to evaluate behavior changes over time [25, 26]. This shorter version of the C-BARQ has previously been used to evaluate dogs at relinquishment [25] and was selected to lower the burden of participation given owners were asked to complete the survey four times, as it has been done elsewhere [19]. Owners scored each behavior on a five-point Likert scale from 0–4. C-BARQ subscales for all four timepoints were compiled based on previously published behavior traits: excitability, stranger-directed aggression, owner-directed aggression, dog-directed aggression, familiar dog aggression, stranger-directed fear, nonsocial fear, dog-directed fear, touch sensitivity, separation-related behavior, attachment and attention-seeking, training difficulty, chasing, and energy level [25, 26].

At each timepoint, owners were also asked about overall satisfaction with their dog’s behavior and household changes since adoption (acquired new pets, moving, and schedule changes). The first survey contained basic demographic and whether they were a first-time pet owner. Finally, at each timepoint, owners were asked if they still owned the dog, and if not, why not. The supplemental (non-CBARQ) questions are included in supplemental materials (S2 File).

Variables obtained from the shelter were evaluated as predictive factors for behavior changes: estimated age, sex, weight, length of stay, intake reason (stray, transfer from another shelter, owner surrender, or seized in a cruelty investigation), medication use, and whether the dog had previously been returned to the shelter. Age (years) was estimated by the shelter staff at the time of intake. Breed was not used in the analysis given the inaccuracy in guessing breeds based on visual appearance since most dogs were listed as mixed [27, 28]. All dogs in the study were neutered before release per shelter policy, so neuter status was also not included in the analysis. The first author (KB) reviewed shelter medical records for each dog to identify which dog was treated with a medication related to a gastrointestinal illness (namely maropitant and metronidazole), antibiotic use (any systemic oral antibiotic to treat an infection), and psychotropic medication use (namely fluoxetine, trazodone, and gabapentin). These were all captured as yes or no. Because of the standard prophylactic use of antiparasitic medications across all shelters, these were not included in the use of gastrointestinal medications.

### Statistical analysis

At the end of the study period, data was exported from RedCap to Excel (Microsoft Office). Entry errors and formatting issues were addressed at this stage and corrected when possible. Statistical analysis was performed using STATA 14. Descriptive statistics were conducted to describe the canine participants and C-BARQ subscales, using means, standard deviation (SD), and proportions depending on the variable types. Multivariable mixed linear models were individually built for each C-BARQ behavior trait (as the dependent variable or outcome). For each model, the main predictor variable of interest was time after adoption (categorical: seven, 30, 90, and 180 days after adoption). Other potential confounders considered in all models included age (continuous, in years estimated by shelter staff at intake), weight (continuous, in pounds), sex (female or male), shelter length of stay (continuous, in days), behavior, GI, and antibiotic medication use in shelter, shelter intake reason, previous adoption (whether the animal had been returned to the shelter or not), and if the owner was a first-time dog owner (yes or no) [19, 29–32]. For attachment/attention-seeking and separation-related problems, additional confounders were included: whether the owner moved during the study period (yes or no) and changes in work schedule during the study period (yes or no) [33]. For the familiar dog aggression model, acquiring a new dog (after the adoption of the survey dog) was included as a confounding variable (yes or no) [34]. Each shelter obtained the previous variables from medical records and sent them to the lead author in a spreadsheet at the end of the study collection period. Since there could be four observations for each dog (one for each survey timepoint) and given that multiple dogs were enrolled from each shelter, both a dog identifier and a shelter identifier were included as random effects (to account for the lack of independence between data points).

Model building occurred in several steps. First, the assumption of linearity between dependent variables and continuous predictors of interest was checked. If not met, predictors were categorized using pre-determined categories from the literature or quartiles. Age categories (< 6 months, 6 months-1.9 years, 2–6.9 years, and ≥ 7 years) were adapted based on a recent review of the developmental stages of dogs [35]. Time in shelter was categorized around author experience of the most useful delineations expanding on previous studies for evaluating short, medium, and long-term shelter stays (0–10 days, 11–30 days, 31–60 days, and >60 days) [36, 37]. The last two categories were lumped together given the very low number of dogs in the shelter greater than 30 days (n = 10). Weight in pounds was categorized as follows: 1–8.9, 9–22.9, 23–55.9, 56–99.9 (0.45–4.0, 4.1–10.4, 10.5–25.4, 25.5–45 kg) [38]. No dogs in the study weighed more than 100 pounds (45kg).

Second, univariable associations between predictors and outcomes were checked individually. Those with P < 0.20 were offered to the multivariable model. For each outcome, the final model was generated using a backwards stepwise approach, with variables being removed from the model one at a time, checking for potential confounding effects. A variable was defined as a confounder when its removal changed another variable’s coefficient by 20% or more and, in this case, confounding variables were retained in the final model regardless of statistical significance. Statistical significance of the overall models was declared at P < 0.05.

Owner satisfaction data is reported descriptively. Prevalence of behavior problems was reported as percent reporting a particular behavior present (> 0 in the C-BARQ subscale).

## Results

Ninety-nine owners completed the initial questionnaire at seven days, 83 completed the questionnaire at 30 days, 75 completed the questionnaire at 90 days, and 83 owners completed the fourth and final survey at 180 days. Sixty-two owners (62.6%) answered all four surveys. Due to a survey coding error in the first half of data collection, if one survey was not completed, subsequent surveys were not sent. This was later fixed so enrolled participants received each questionnaire regardless of completion of previous surveys.

Most owners (62.9%) had owned a dog in the past. There were vastly more female respondents (75.0%) compared to male (24.0%), with 1.0% in neither category. The population was about evenly split between who had other animals in the house (52.5%) versus not (47.5%) prior to adopting the survey dog. Household characteristics are summarized in Table 1. Table 2 reviews the dog demographic and shelter data for the study population. Seven people returned their adopted dog during the study period (a return rate of 7.1%). The prevalence of undesirable behavior based on C-BARQ subscales are reported in Table 3.

**Table 1:** Household characteristics (*n* = 99).

Variable	Mean	Std. Dev.
Average household size (# of people)	2.6	1.35
Average age of adopter	34.6	13.3
Variable	N	%
**First time dog owner?**		
Yes	37	38.1%
No	60	61.9%
**Other animals in home at 10 days?**		
No	47	47.5%
Yes	52	52.5%
**What other animals?**		
Dogs	38	39.2%
Cats	28	28.9%
Other animals	12	12.4%
**Highest degree obtained**		
No diploma or degree obtained	0	0.0%
High school graduate, diploma or equivalent (GED)	22	22.7%
Trade/technical/vocational training	5	5.2%
Associate degree	9	9.3%
Bachelor’s degree	37	38.1%
Master’s degree	13	13.4%
Professional/Doctorate degree	10	10.3%
Other?	1	1.0%
**Gender**		
Female	72	75.0%
Male	23	24.0%
Other/Prefer to self-describe	1	1.0%
Prefer not to disclose	0	0.0%
**Household Income**		
Less than $24,999	10	10.4%
$25,000 - $49,999	17	17.7%
$50,000 - $74,999	15	15.6%
$75000 - $99,999	13	13.5%
$100,000 - $124,999	16	16.6%
$125,000 - $149,999	6	6.2%
$150,000 and over	8	8.3%
Prefer not to answer	11	11.4%
**Race**		
American Indian or Alaska Native	1	1.0%
Asian	6	6.2%
Black or African American	3	3.0%
Hispanic or Latino	2	2.0%
White	82	84.5%
Prefer not to answer	3	3.0%
**Home Type**		
Apartment/Condo	36	37.1%
Mobile Home	1	1.0%
Single-family Home	56	57.7%
Farm	3	3.0%
Other	1	1.0%

**Table 2:** Dog characteristics (*n* = 99).

Variable	N (%)
Sex	
Male	47 (47.5%)
Female	52 (52.5%)
Age (mean)	3.0 years (SD 2.30)
Age Categories	
<6m	7 (7.2%)
6m - 2y	9 (9.3%)
2-7y	54 (55.7%)
>7y	8 (8.2%)
Weight (mean)	41.7 pounds (SD 18.62)
Weight Categories	
<9#	5 (5.1%)
9–23#	14 (14.3%)
23–56#	57 (58.2%)
56–100#	22 (22.4%)
Prescribed antibiotics in shelter	
Yes	17 (17.5%)
No	80 (82.5%)
Prescribed gastrointestinal medications in shelter	
Yes	4 (4.1%)
No	93 (95.9%)
Prescribed behavioral medications in shelter	
Yes	34 (35.1%)
No	63 (65.0%)
Intake Status	
Owner surrender	14 (14.1%)
Cruelty/neglect case	4 (4.0%)
Stray	49 (49.5%)
Transfer	30 (30.3%)
Days in shelter (mean)	14.8 days (SD 15.7)
Days in shelter	
0–10 days	53 (55.8%)
11–30 days	32 (33.7%)
>30 days	10 (10.5%)
Dog previously returned before study?	
Yes	7 (7.8%)
No	90 (92.8%)
Dog returned after starting study?	
Yes	7 (7.1%)
No	92 (93.0%)
Days in shelter	
Median	10 (SD 15.68)
Mean	14.8

Characteristics of the dog population are presented. Gastrointestinal medications including anything to treat diarrhea or vomiting (namely metronidazole or maropitant, but not antiparasitic medication due to their ubiquitous use), antibiotic use (any systemic oral antibiotic to treat an infection), and psychotropic medication use (e.g. fluoxetine, trazodone, and gabapentin).

**Table 3:** Prevalence of undesirable behaviors based on C-BARQ subscales.

C-BARQ subscales	10 days		30 days		90 days		180 days	
	n	%	n	%	n	%	n	%
**Stranger-directed aggression**	**56**	**61.5%**	**52**	**66.7%**	**58**	**81.7%**	**60**	**76.9%**
Owner-directed aggression	31	32.3%	20	25.0%	21	30.0%	22	27.2%
Dog-directed aggression	54	62.8%	56	74.7%	48	66.7%	60	75.0%
Familiar dog aggression	17	32.7%	15	35.7%	10	25.6%	17	37.8%
Stranger-directed fear	43	46.7%	46	58.2%	34	47.2%	44	55.0%
Nonsocial fear	87	89.7%	75	92.6%	69	95.8%	69	86.3%
Dog-directed fear	58	71.6%	60	80.0%	54	76.1%	53	67.1%
**Separation-related behaviors**	**81**	**92.0%**	**69**	**89.6%**	**63**	**92.6%**	**63**	**87.5%**

Data were treated as binary variables (a score of 0 = no present; a score of >0 = present). Missing values were omitted so the denominator does not always equal the total number of people taking the survey at each timepoint. Of these reported behaviors, only stranger-directed aggression and separation-related behaviors were found to change significantly for at least one timepoint throughout the study (bolded).

Based on the final models, the following C-BARQ traits changed significantly from baseline C-BARQ (seven days after adoption):

1. Stranger-directed aggression–Increases reported at all time points
2. Excitability–Increases reported at 90 and 180 days
3. Touch sensitivity–Increases reported at 90 and 180 days
4. Chasing behavior–Increases reported at all time points
5. Training Difficulty–Increases reported at all time points
6. Separation-related behaviors–Decrease reported at 180 days
7. Attachment and attention-seeking–Decrease reported at 180 days

**Fig 1:**
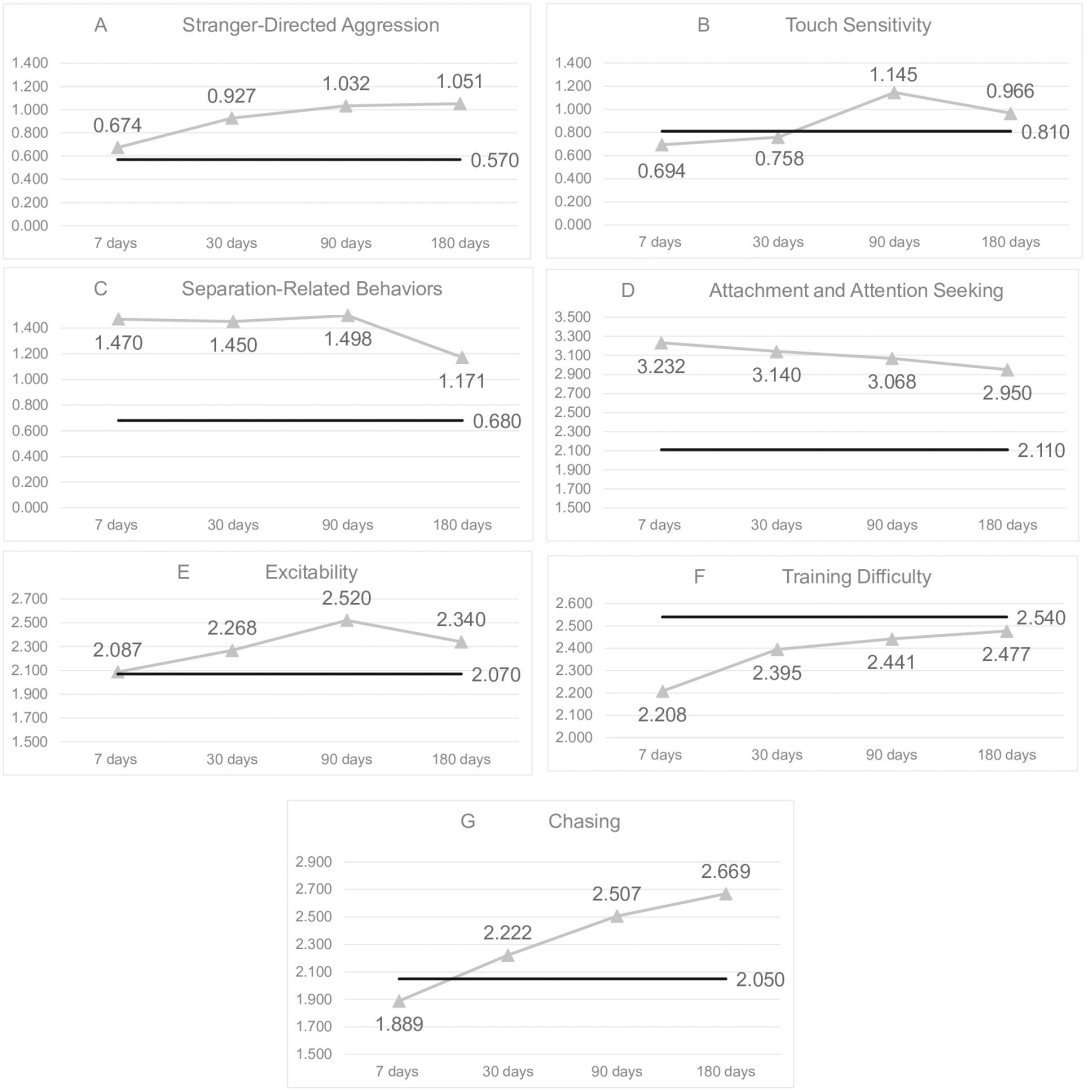
C-BARQ scores compared to national averages.

There were no significant differences throughout the study period for the remaining C-BARQ subscales: familiar dog aggression, owner-directed aggression, dog-directed aggression, stranger-directed fear, nonsocial fear, dog-directed fear, or energy level. Final models are shown in Tables 4–10. Additionally, Fig 1(A)–1(G) shows C-BARQ scores at each timepoint for the seven behaviors that significantly changed compared to national C-BARQ averages.

**Table 4:** Final mixed linear model for C-BARQ subscale stranger-directed aggression (*n* = 95).

Variable	Coefficient	Std. Err.	95% CI	P value
Stranger-directed aggression scores				
7 days	Reference	-	-	-
**30 days**	**0.25**	**0.08**	**(0.08, 0.42)**	**0.005** ***
**90 days**	**0.44**	**0.09**	**(0.26, 0.61)**	**<0.001** ***
**180 days**	**0.44**	**0.09**	**(0.27, 0.61)**	**<0.001** ***
Intake Status				
Cruelty/Neglect	Reference	-	-	-
Owner Surrender	0.42	0.42	(-0.40, 1.24)	0.314
Transfer	-0.12	0.39	(-0.89, 0.64)	0.752
Stray	0.07	0.38	(-0.67, 0.81)	0.860
Age				
<6m	Reference	**-**	**-**	**-**
6m - 2y	0.75	0.38	(0.00, 1.50)	0.050*
2-7y	0.71	0.38	(-0.04, 1.46)	0.064*
>7y	0.38	0.44	(-0.47, 1.23)	0.382
Weight				
<9#	Reference	**-**	**-**	**-**
**9–23#**	**1.05**	**0.40**	**(0.26, 1.84)**	**0.009** ***
23–56#	0.52	0.37	(-0.21, 1.24)	0.163
56–100#	0.23	0.40	(-0.55, 1.02)	0.563
Behavior Medications in shelter				
No	Reference	**-**	**-**	**-**
**Yes**	**0.41**	**0.17**	**(0.09, 0.74)**	**0.013** **
Model Constant	1.24	0.43	(0.41, 2.09)	0.004

A multivariable mixed linear model was built controlling for shelter, age, weight, and behavior medication use in the shelter. An increase in stranger-directed aggression was significant at all three timepoints compared to baseline. In the final model, dogs weighing between 9–22.9 pounds and behavior medication use in the shelter were also positive predictors of stranger-directed aggression. Dogs between 6 months to 2 years and 2 years to 7 years of age trended toward a positive predictor of stranger-directed aggression.

*Statistical significance at p<0.10;

**Statistical significance at p<0.05;

***Statistical significance at p<0.01

Variables with p-values < 0.05 are bolded.

**Table 5:** Final mixed linear model for C-BARQ subscale excitability (*n* = 95).

Variable	Coefficient	Std. Err.	95% CI	P value
Excitability scores				
7 days	Reference	-	-	-
**30 days**	0.12	0.12	(-0.11, 0.35)	0.307
**90 days**	**0.42**	**0.12**	**(0.19, 0.66)**	**<0.001** ***
**180 days**	**0.27**	**0.12**	**(0.04, 0.50)**	**0.021** **
Intake Status				
Cruelty/Neglect	Reference	-	-	-
Owner Surrender	0.17	0.50	(-0.81, 1.14)	0.739
Transfer	0.55	0.45	(-0.33, 1.42)	0.220
Stray	0.44	0.46	(-0.46, 1.35)	0.338
Days in shelter				
0–10 days	Reference	**-**	**-**	**-**
11–30 days	0.23	0.19	(-0.14, 0.61)	0.220
>30 days	-0.58	0.32	(-1.21, 0.04)	0.065*
Sex				
Male	Reference	**-**	**-**	**-**
Female	-0.33	0.18	(-0.68, 0.09)	0.064*
Behavior Medications in shelter				
No	Reference	**-**	**-**	**-**
Yes	0.32	0.20	(-0.07, 0.70)	0.108
Model Constant	1.65	0.46	(0.74, 2.56)	<0.001

A multivariable mixed linear model was built controlling for shelter, days in shelter, sex, and behavior medication use in the shelter. An increase in excitability was significant at 90 and 180 days compared to baseline. In the final model, >30 days in the shelter and being female trended towards higher excitability scores.

*Statistical significance at p<0.10;

**Statistical significance at p<0.05;

***Statistical significance at p<0.01

Variables with p-values < 0.05 are bolded.

**Table 6:** Final mixed linear model for C-BARQ subscale touch sensitivity (*n* = 83).

Variable	Coefficient	Std. Err.	95% CI	P value
Touch sensitivity scores				
7 days	Reference	-	-	-
30 days	0.07	0.13	(-0.18, 0.33)	0.578
**90 days**	**0.41**	**0.13**	**(0.15, 0.67)**	**0.002** ***
**180 days**	**0.25**	**0.13**	**(0.00, 0.50)**	**0.049** **
Intake Status				
Cruelty/Neglect	Reference	-	-	-
Owner Surrender	0.42	0.42	(-0.40, 1.24)	0.314
Transfer	-0.12	0.39	(-0.89, 0.64)	0.752
Stray	0.07	0.38	(-0.67, 0.81)	0.860
Days in shelter				
0–10 days	Reference	**-**	**-**	**-**
11–30 days	0.09	0.15	(-0.21, 0.39)	0.551
>30 days	-0.28	0.25	(-0.77, 0.20)	0.254
Weight				
<9#	Reference	**-**	**-**	**-**
9–23#	0.54	0.43	(-0.30, 1.39)	0.207
23–56#	0.40	0.39	(-0.38, 1.17)	0.305
56–100#	0.21	0.41	(-0.60, 1.04)	0.606
Behavior Medications in shelter				
No	Reference	**-**	**-**	**-**
**Yes**	**0.42**	**0.15**	**(0.12, 0.72)**	**0.005** ***
First-time dog owner				
No	Reference	**-**	**-**	**-**
Yes	0.24	0.15	(-0.06, 0.53)	0.116
Model Constant	0.13	0.39	(-0.64, 0.90)	0.741

A multivariable mixed linear model was built controlling for shelter, days in shelter, weight, behavior medication use in the shelter, and first-time dog ownership. An increase in touch sensitivity was significant at 90 and 180 days compared to baseline. In the final model, behavior medication use in the shelter was also a positive predictor of touch sensitivity.

*Statistical significance at p<0.10;

**Statistical significance at p<0.05;

***Statistical significance at p<0.01

Variables with p-values < 0.05 are bolded.

**Table 7:** Final mixed Linear Model for C-BARQ subscale chasing (*n* = 95).

Variable	Coefficient	Std. Err.	95% CI	P value
Chasing scores				
7 days	Reference	-	-	-
**30 days**	**0.24**	**0.12**	**(0.00, 0.46)**	**0.041** **
**90 days**	**0.56**	**0.12**	**(0.33, 0.79)**	**<0.001** ***
**180 days**	**0.64**	**0.12**	**(0.41, 0.86)**	**<0.001** ***
Age				
<6m	Reference	**-**	**-**	**-**
6m - 2y	-0.15	0.46	(-1.05, 0.76)	0.751
2-7y	0.42	0.45	(-0.46, 1.31)	0.349
>7y	0.06	0.53	(-0.398, 1.11)	0.908
Behavior Medications in shelter				
No	Reference	**-**	**-**	**-**
Yes	0.37	0.21	(-0.05, 0.79)	0.083*
Model Constant	1.61	0.43	(0.78, 2.45)	<0.001

A multivariable mixed linear model was built controlling for age and behavior medication use in the shelter. An increase in chasing was significant at all three timepoints compared to baseline. In the final model, behavior medication use in the shelter was also a positive predictor of chasing.

*Statistical significance at p<0.10;

**Statistical significance at p<0.05;

***Statistical significance at p<0.01

Variables with p-values < 0.05 are bolded.

**Table 8:** Final mixed linear model for C-BARQ subscale training difficulty (*n* = 96).

Variable	Coefficient	Std. Err.	95% CI	P value
Training Difficulty Scores				
7 days	Reference	-	-	-
**30 days**	**0.19**	**0.07**	**(0.05, 0.32)**	**0.007** ***
**90 days**	**0.23**	**0.07**	**(0.09, 0.37)**	**0.001** ***
**180 days**	**0.28**	**0.07**	**(0.14, 0.42)**	**<0.001** ***
Intake Status				
Cruelty/Neglect	Reference	-	-	-
Owner Surrender	0.07	0.23	(-0.38, 0.52)	0.765
Transfer	0.39	0.22	(-0.04, 0.82)	0.074*
Stray	0.31	0.21	(-0.10, 0.72)	0.142
Age				
<6m	Reference	**-**	**-**	**-**
6m - 2y	-0.27	0.22	(-0.70, 0.15)	0.211
2-7y	-0.29	0.21	(-0.71, 0.13)	0.178
**>7y**	**-0.57**	**0.25**	**(-1.06, -0.09)**	**0.02** **
Weight				
<9#	Reference	**-**	**-**	**-**
9–23#	-0.01	0.24	(-0.48, 0.45)	0.951
23–56#	0.31	0.22	(-0.12, 0.74)	0.157
**56–100#**	**0.53**	**0.24**	**(0.06, 1.00)**	0.026**
Model Constant	1.93	0.30	(1.33, 2.52)	<0.001

A multivariable mixed linear model was built controlling for shelter, age, and weight. An increase in training difficulty was significant at all three timepoints compared to baseline. In the final model, dogs weighing between 50–100 pounds was a positive predictor of trainability. There was a significant negative association between dogs >7 years and training difficulty.

*Statistical significance at p<0.10;

**Statistical significance at p<0.05;

***Statistical significance at p<0.01

Variables with p-values < 0.05 are bolded.

**Table 9:** Final mixed linear model for C-BARQ subscale separation-related problems (*n* = 94).

Variable	Coefficient	Std. Err.	95% CI	P value
Separation-related problem scores				
7 days	Reference	-	-	-
30 days	-0.03	0.11	(-0.24, 0.19)	0.805
90 days	0.04	0.11	(-0.18, 0.26)	0.730
**180 days**	**-0.23**	**0.11**	**(-0.45, -0.02)**	**0.034** **
Age				
<6m	Reference	**-**	**-**	**-**
6m - 2y	-0.51	0.36	(-1.22, 0.20)	0.158
**2-7y**	**-0.72**	**0.36**	**(-1.42, -0.01)**	**0.046** **
>7y	-0.73	0.41	(-1.54, 0.07)	0.073*
Weight				
<9#	Reference	**-**	**-**	**-**
9–23#	0.53	0.39	(-0.24, 1.29)	0.177
23–56#	52.00	0.36	(-0.19, 1.22)	0.149
56–100#	0.52	0.40	(-0.25, 1.30)	0.183
Behavior Medications in shelter				
No	Reference	**-**	**-**	**-**
Yes	0.26	0.16	(-.05, 0.56)	0.099*
Sex				
Male	Reference	**-**	**-**	**-**
Female	0.30	0.15	(0.00, 0.60)	0.051*
Model Constant	1.24	0.43	(0.41, 2.09)	0.004

A multivariable mixed linear model was built controlling for age, weight, behavior medication use in the shelter, and sex. An increase decrease in separation-related problems was significant at 180 days compared to baseline. In the final model there dogs between two and seven years of age were less likely to show separation related behaviors. There were also trends towards increased separation-related problems with behavior medication use in the shelter and being female.

*Statistical significance at p<0.10;

**Statistical significance at p<0.05;

***Statistical significance at p<0.01

Variables with p-values < 0.05 are bolded.

**Table 10:** Final mixed linear model for C-BARQ subscale attachment and attention-seeking (*n* = 97).

Variable	Coefficient	Std. Err.	95% CI	P value
Attachment and attention-seeking scores				
7 days	Reference	-	-	-
30 days	-0.08	0.08	(-0.23, 0.07)	0.276
90 days	-0.15	0.08	(-0.30, 0.01)	0.062*
**180 days**	**-0.31**	**0.08**	**(-0.46, -0.16)**	**<0.001** ***
Intake Status				
Cruelty/Neglect	Reference	-	-	-
Owner Surrender	0.52	0.32	(-0.11, 1.15)	0.107
Transfer	0.10	0.30	(-0.49, 0.69)	0.74
Stray	0.42	0.30	(-0.15, 1.00)	0.151
Model Constant	2.95	0.29	(2.39, 3.51)	<0.001

A multivariable mixed linear model was built controlling for shelter. An decrease in attachment and attention-seeking behavior was significantly decreased at 180 days compared to baseline. There was a trend towards lower scores at 90 days as well.

*Statistical significance at p<0.10;

**Statistical significance at p<0.05;

***Statistical significance at p<0.01

Variables with p-values < 0.05 are bolded.

Each C-BARQ subscale found to significantly change after adoption is graphed and compared to the national C-BARQ average for that subscale. The national C-BARQ averages were obtained through personal communication with Dr. James A. Serpell, University of Pennsylvania C-BARQ Project). They represent USA data as of 03-17-2022 for breeds with >30 entries (including mixed breeds), which is included in supplemental information (S3 File). A solid black line represents the national C-BARQ average, whereas the grey line with triangle markers represents the study population C-BARQ scores at 7, 30, 90, and 180 days.

At 180 days, 100% of owners indicated their dog adjusted to the new home extremely or moderately well, with no owner indicating poorly/not at all. Also at 180 days, 93.7% rated their dog’s overall behavior as excellent or good, 6.3% as fair, and no owner reported poor/terrible. Seventy-five percent of owners indicated their dog’s overall behavior improved by 180 days, with 21.2% reported it stayed the same, and 3.8% reported worsening behavior. The owner satisfaction data is summarized for each timepoint in Table 11.

**Table 11:** Overall behavior satisfaction (*n* = 99).

Variable	10 days	30 days	90 days	180 days
Overall, how well do you feel that your dog is adjusting to its new home?				
Extremely well	73 (75.5%)	64 (75.3%)	60 (82.2%)	65 (83.3%)
Moderately well	21 (21.4%)	17 (20.0%)	13 (17.8%)	13 (16.7%)
Fair	4 (4.1%)	4 (4.7%)	0 (0%)	0 (0%)
Poorly/Not at all	0 (0%)	0 (0%)	0 (0%)	0 (0%)
Overall, how do you feel your dog’s behavior has changed since adoption?				
Improved	70 (72.2%)	56 (68.3%)	54 (74.0%)	60 (75.0%)
Stayed about the same	24 (24.7%)	22 (26.8%)	18 (24.7%)	17 (21.2%)
Worsened	3 (3.1%)	4 (4.9%)	1 (1.4%)	3 (3.8%)
Overall, how would you describe your dog’s behavior?	0%			
Excellent	-	25 (29.4%)	25 (32.9%)	30 (38.0%)
Good	-	55 (64.7%)	45 (59.2%)	44 (55.7%)
Fair	-	4 (4.7%)	6 (7.9%)	5 (6.3%)
Poor/Terrible	-	1 (1.2%)	0 (0%)	0 (0%)

Owners were asked to report their overall impression of their dog’s behavior and how their behavior has changed at each timepoint. Overall owners reported very high ratings of the behavior of their adopted dogs, and most reported it has improved over the study period.

## Discussion

The goal of this study was to identify behavior changes in dogs adopted from shelters over the first 180 days (six months) in their new home. The use of a standardized questionnaire for owners and tracking individual dog behavior over multiple timepoints are strengths of this study. Previous investigations of post-adoption dog behavior have largely relied on cross-sectional data at a single time point and/or ad hoc surveys, with one recent exception [19]. Additionally, the current literature primarily tracks dog behavior outside the United States [11, 12, 15, 17, 18, 20, 39–41]. These dogs are obtained and managed much differently than dogs in the United States (such as differing neuter status, housing, and tolerance for roaming) so generalizing results from studies conducted in other countries is challenging [42].

Survey data generally result in high satisfaction for adopted dogs, despite behavior problems, as people tend see their dog through rose-colored glasses [16, 17, 19, 20, 39, 40]. Most of the findings here represented a worsening of behavior over time (stranger-directed aggression, excitably, touch sensitivity, training difficulty, and chasing behavior) versus an improvement (separation-related behaviors). Attachment and attention-seeking changes, which decreased, could be viewed as improvement or worsening, depending on the owner and circumstance, so their influence on satisfaction is likely mixed. Despite these findings, as with previous studies, the vast majority of owners rated their new dog’s behavior as excellent or good (93%). About 75% of owners described their dog’s behavior as improved throughout the study period, indicating they may prioritize behaviors that improved (separation-related problems) over other behaviors that may have worsened. Given the small magnitude of improvement in separation-related problems, this is unlikely. Some behaviors are more easily avoidable or tolerable depending on the household. Behaviors such as chasing small wild animals is a normal behavior in dogs that owners may not perceive as a significant problem for the household or dog. There also may be behaviors the C-BARQ is not capturing, but still highly valued by owners, that reflect improvement in the human-animal bond as dogs adjust to their new home. Further evaluation on this point should be prioritized in future research.

An important finding in this study is the increase from baseline in stranger-directed aggression at all three timepoints. Canine aggression directed to people represents a significant public health concern, with more than 4.5 million bites in the U.S. annually [43]. Of the reasons cited for relinquishing a dog to a shelter, behavioral concerns, namely aggression, are at the top [10, 19, 44]. The coefficient represents almost a half point increase in the C-BARQ subscale at 180 days from baseline. This change may appear small on an individual dog level, but if extrapolated to the general dog population, could represent a sizable portion of human directed aggression in the U.S. In addition to the increased stranger-directed aggression throughout the study, prevalence was also notably high with 76.9% of dogs showing at least some level of stranger-directed aggression at 180 days [45, 46]. Data is lacking on the overall prevalence of stranger-directed aggression in dogs and primarily focuses on the prevalence of dog bites, but has been estimated previously to occur in 5 to 9.2% of dogs [45, 46].

In previous studies, male dogs have shown high rates of stranger-directed aggression but this was not repeated in this study [45–51]. Use of behavior medications in the shelter was associated with higher stranger-directed aggression scores post adoption. This finding is not surprising as the use of behavior medications in the shelter is often triggered by aggressive behavior during veterinary handling. The staff at shelters are almost always strangers to the dog, at least during the initial part of the dog’s stay when the bulk of handling typically occurs.

The difference between these studies and the current data may be partly explained by different methodologies for defining aggression (not limiting our data to just bites), but also geographic or temporal differences. This study data was obtained during the COVID-19 pandemic, occurring largely during a time of strict lockdowns. Social distancing and altered human behaviors may have increased certain problematic canine behaviors. Additionally, some of the items in the stranger-directed aggression subscale address territorial behavior, which would be expected to increase over time as the dog settles into their new home.

There was a very high prevalence of separation-related behaviors reported in the study (87.5% at 180 days), with a small but statistically significant decrease between 90 and 180 days. A recent study also using C-BARQ as their survey tool for post-adoption behavior found a similarly high prevalence (79.2% - 85.5%), with the severity increasing between two days and four months, but not statistically significant [19]. Previous studies have found separation-related behaviors in a much lower percent of their sample populations (5% to 29%) [52–57]. The two most recent studies with higher incident rates occurred at least partially through the COVID-19 pandemic. Pandemic-associated, severe schedule changes (home all the time, versus back to work/school) may have contributed to the development of separation anxiety [58, 59]. While statistically significant, the decrease in reported separation-related behaviors at 180 days was small (0.23 points of the C-BARQ score). However, this information is still potentially helpful for owners whose dogs show signs of separation-related problems as there may be a small, but naturally occurring decrease as the dog gets more comfortable at home after 3 months. This data provides evidence that a high proportion of dogs experience separation-related problems after adoption but some improve. It may take tracking this trend and home-life variables for a greater length of time to evaluate whether additional dogs improve over time.

Attachment and attention-seeking behavior (following the owner around the house and sitting near the owner) followed a similar pattern to separation-related behaviors, decreasing mostly significantly between 90 and 180 days. This suggests dogs may gain confidence and a sense of security over time and rely less on proximity to people as they gain comfort in their new home. Additionally, dogs may learn their owner’s predictable schedule so there is less attention seeking required to meet their needs.

Chasing behavior (chasing birds, squirrels, rabbits, etc.) showed the largest numerical increases in scores over the study period and increased at each timepoint compared to baseline (0.64 increase at 180 days compared to baseline). This contrasts with the other recent study using C-BARQ tracking dog behavior after adoption which found no significant differences in chasing behavior between two days and four months [19]. Chasing behavior is a normal manifestation of the predatory instinct in dogs and experts agree that it generally entails a neutral or positive affective state [60–62]. There are breed differences in the prevalence of predatory behavior in dogs which may explain the differences from studies [63]. Since most of the dogs in this study were mixed breed, breed was not included in the analysis thus it is impossible to draw any definitive conclusions. It is unclear why there was a large increase in chasing behaviors over time. Owners may give dogs more freedom as they become more comfortable with their dog’s behavior overall, thus giving dogs more opportunities to chase small animals. Because chasing small animals is a normal and expected behavior of dogs, these increases may also not be important to dog owners.

Touch sensitivity (nail trimming, bathing, and grooming) increased at days 90 and 180, with the most significant increase (P< 0.01) reported at 90 days (decrease in 0.41 points of the C-BARQ subscale). This may simply represent the natural timeline of when owners attempt to trim their dogs’ nails and bathe/groom them after adoption, as many dogs are adopted out recently bathed and with trimmed nails after spay/neuter surgeries. Behavior medication use was also significant in this model which is logical given dogs who are more sensitive to touch may be more difficult to examine by veterinary staff or groom in the shelter, thus necessitating oral anxiolytic medications to reduce stress related to handling. Knowing that the need for this in the shelter is predictive of increased sensitivity in the home is critical information to relay to adopters.

Excitability increased at both 90 and 180 days and was associated with longer shelter stays. Consistent with this finding, previous research has shown increased time lying down during the dog-adoptor interaction was associated with shorter length of stays, whereas back and forth movements in the kennel were associated with longer length of stays [64, 65]. Increased excitability may also negatively impact owner attachment to their dog, increase owner frustration, and become an important factor for relinquishment [66, 67]. The association between excitability and relinquishment was not replicated in this study given low relinquishment rates.

Training difficulty increased at all timepoints throughout the study. Trainability has previously been associated with increased owner attachment and attachment scores decreased slightly throughout our study. Apart from the decrease in attachment scores, it is unclear why training difficulty increased through all time points. There may be more opportunities as time progresses after adoption for owners to truly assess their new dog’s behavior in a training setting. Dogs may also be behaviorally blunted, as discussed before, right after adoption and thus remain in a sit/stay for longer and appear less distracted. Our study disagrees with previous findings that older dogs are less trainable, at least as reported by owners [68–70]. Dogs >7 years of age had significantly decreased training difficulty (P ≤ 0.05). It has been demonstrated that olderolder dogs can still readily learn new behaviors and perform well in a training class [71]. Older dogs have more life experience, and thus may respond more consistently to human training cues. Also, dogs greater than 56 pounds (25.5kg) scored significantly higher on training difficulty scores (P ≤ 0.05).

Seven percent of the dogs in this study were previously returned prior to their most recent adoption with also 7% returned after enrolling in the study. This is about half the reported average return rates for dogs of approximately 15% in the USA [72].

While dog ownership has been lauded for its many benefits during a time of increased social isolation during to the global COVID-19 pandemic, there are notable outliers and exceptions to this general trend [59, 73, 74]. Early research on the effects of dog ownership during the COVID-19 pandemic has noted benefits for owner mental health, but also significant challenges for owners, including new behavior problems, balancing dogs needs with public health guidance, and accessing supplies and services for their dog [59, 74]. In one study, the top concern for people managing a pet during the pandemic was meeting their “social and behavioral needs due to changes in everyday life from the pandemic” [59]. Missed or limited training opportunities was also a cited concern [59]. Owners reported fewer walks during strict lockdowns and decreased interactions with other people, which may have limited exposure and socialization opportunities for newly acquired dogs [74]. Additionally, dog bites to children skyrocketed during lockdowns [75, 76]. A full appreciation of the role of COVID-19 related policies, household changes, and stressors will require a comparison study with a similar population during non-pandemic times. Given the current body of literature, it can be strongly inferred that the global pandemic likely had an impact on this population.

This study has several limitations, including geographic bias (five shelters in two Ohio cities). There is likely sampling bias: owners who are more pleased with their dog’s behavior may be more likely to take the time to fill out the questionnaire, especially given the low relinquishment rates compared to national trends, but the magnitude and direction of bias is difficult to assess, as the opposite could also be true. We had far more female owners participate in the study than males, but this is consistent with other studies [77]. Women are also more likely in general to participate in the care of pets [78]. While the shortened versions of the C-BARQ behavior subscales are highly correlated with the longer 100 question subscales, the shortened C-BARQ is not formally validated. This study did not investigate the role treatment had in influencing behavior changes outlined in this study (or lack thereof).

This comprehensive prospective study using multivariate analysis, multiple timepoints, and a validated tool to track dog behavior adds to the current body of literature on canine post-adoption behavior. These results provide veterinarians, canine behavior professionals, and shelter staff a better understanding of the post-adoption behavior of dogs adopted from a shelter setting across the first six months after adoption. Owners can be counseled about what behavior changes to expect and when. By providing owners with accurate information on what behavior changes to expect, owners may have more realistic expectations on their dog’s future behavior. This may mean fewer dogs rehomed, returned, or euthanized, advancing canine health and welfare. Additionally, this data guides shelters on what interventions may be helpful, and when, to keep dogs in their adoptive homes.

## Supporting information

S1 File Shelter script. (DOCX)

S2 File Additional questions apart from C-BARQ. (DOCX)

S3 File National C-BARQ breed and all-breed means from USA. (XLSX)

S4 File Complete survey response data: All C-BARQ data used for data analysis in the study. (CSV)

S5 File Shelter dog demographic data: Spreadsheet of all the demographic data for the dogs in the study. (XLSX)

S6 File Endnotes. (DOCX)
