# Supplementary material for: Shelter dog behavior after adoption: Using the C-BARQ to track dog behavior changes through the first six months after adoption
Source: PLoS One. 2023 Aug 16;18(8):e0289356. doi: 10.1371/journal.pone.0289356 (PMC10431636; doi:10.1371/journal.pone.0289356)
Supplement: S1 File — (DOCX) [file pone.0289356.s001.docx]

Shelter dog behavior after adoption: Using the C-BARQ to track dog behavior changes through the first six months after adoption

**Supplemental Material**

Shelter prompt for discussing study with adopters:

“*This is information about a research study from The Ohio State University College of Veterinary Medicine tracking dog behavior after adoption. Please see this flier for additional information and reach out to the research team with any questions.*”

Additional questions apart from C-BARQ:

As of today, do you currently own the dog you adopted from the shelter?

Yes

No

Was your dog:

- Returned to the shelter
- Rehomed to a new owner
- Lost/stolen/ran away
- Relinquished to a different shelter or rescue
- Sold to a new owner
- Died
- Euthanized
- Other
- Not applicable

Since acquiring your dog from the shelter, have any of the following changes occurred in your household? Check all that apply.

- Acquired another new dog
- Acquired a cat
- Housemate dog (not your recently adopted dog) removed from the house (passed away, given away, lost,
- etc)
- New person (adult) moved in with you
- New person (child) moved in with you
- Had a baby
- Moved
- Started working outside the home after a period of working from home
- Other major changes in the household (layoff, new work schedule, home construction, etc)
- Not applicable

Define other major changes:

Overall, how well do you feel that your dog is adjusting to its new home?

Extremely well, moderately well, fair, poorly/not at all

Overall, how do you feel your dog’s behavior has changed since adoption?

Improved, stayed about the same, worsened

Overall, how would you describe your dog’s behavior?

Excellent, good, fair, poor/terrible

**Demographic/owner characteristics**

Is this the first dog you have owned since becoming an adult (reaching 18 years of age)?

What is your age? (fill in)

How many people live in your household, including yourself?

________

Please list their ages:

What type of home do you live in?

Apartment/condo, mobile home, single-family home, farm

What is the highest degree or level of school you have completed? *If currently enrolled, highest degree received.*

No diploma or degree obtained

High school graduate, diploma or equivalent (for example: GED)

Trade/technical/vocational training

Associate’s degree

Bachelor’s degree

Master’s degree

Professional/Doctorate degree

What is your gender?

Female

Male

Other/prefer to self-describe (fill in)

Prefer not to disclose

What is your household annual income?

Less than $24,999

$25,000 – $49,999

$50,000 – $74,999

$75,000 – $99,999

$100,000 – $124,999

$125,000 – 150,000

$150,000 and over

Prefer not to answer

What is your race or ethnic category?

American Indian or Alaska Native

Asian

Black or African American

Hispanic or Latino

Native Hawaiian or Other Pacific Islander

White

Other_______

Prefer not to answer
