# Supplementary material for: Shelter dog behavior after adoption: Using the C-BARQ to track dog behavior changes through the first six months after adoption
Source: PLoS One. 2023 Aug 16;18(8):e0289356. doi: 10.1371/journal.pone.0289356 (PMC10431636; doi:10.1371/journal.pone.0289356)
Supplement: S6 File — (DOCX) [file pone.0289356.s006.docx]

**Endnotes**

^a^C-BARQ is maintained by the University of Pennsylvania School of Veterinary Medicine, Philadelphia, PA 19104, USA

^b^REDCap is maintained by Vanderbilt University, Nashville, TN 37235, USA

^c^Amazon Incorporated, Seattle, WA 98109, USA

^d^Microsoft Corporation, Redmond, WA 98052, USA

^e^StataCorp LP, College Station, TX 77845, USA
